# Trismus Nascentium

**Published:** 1846-08

**Authors:** 


*Trismus Nascentium.*—The editors of the Southern Medical and Surgical Journal, for July, noticing Dr. Sims's plan of preventing trismus nascentium by providing the infant with a soft pillow, say, "We have given the above directions a fair trial, and like every other treatment for lock-jaw in infants, we have to write—'*Tekel.*'" On the same subject the N. O. Medical Journal contains the following:

[In compliance with the call made in our January number, for information concerning this fatal disease, we have been favored with the following letters, which we take pleasure in laying before our readers. We have but little doubt that the cause of the disease is intimately connected with the management of the umbilicus, and therefore think our correspondent's suggestion valuable. In connection with this subject we will mention, that a Creole professional friend recently informed us, that *Trismus Nascentium* was much more common amongst the plantations below the city a few years ago, than it is now; and that the present exemption is attributed to a plan of managing the navel adopted within the last few years: it consists simply *in dressing it with balsam copaiba.* We learn, that since the adoption of this, the disease is scarcely known in that neighborhood. We should be pleased to receive farther facts and suggestions on the subject.—We had expected to insert Dr. Fitzhugh's communication in our last number, but it was unavoidably postponed. We now have the pleasure of adding Dr. Wooten's interesting letter on the same subject, which has arrived just in time for the press. Dr. W. seems to doubt the correctness of the opinion we expressed in our last number, that this disease is more common in this city than any where else in the United States. Our remark was solely based upon the large number of cases found among our bills of mortality. We should be pleased to receive farther facts and observations in relation to its prevalence throughout the southern States, and especially as to its comparative frequency among white and black infants. The disease certainly occurs among both classes in this city, but to what relative extent we are not prepared to state at present. We repeat our invitation for farther facts.—Edts.]

*Messrs. Editors:* Having observed in your valuable journal, the great mortality in your city from *Trismus Infantum*, or *Tetanus Nascentium*, I am induced to offer the following view of its cause.

I have seen three cases only, all of which proved fatal. The symptoms were precisely those stated by Dr. Eberle in your January number; I therefore deem it unnecessary to repeat them.

The treatment which I adopted in these cases was somewhat different in each. I, however, blistered the abdomen, gave calomel, tinct. opii, oil, &c., without any good effect whatever. I doubt not, if taken in time, the treatment recommended by Dr. Eberle would be very efficient.

It is the cause of this alarming disease, however, which I wish to consider. Believing it proceeded from some mismanagement in dressing the navel, I requested the owner of that plantation to let me know when the next accouchment took place. He did so. I attended particularly to the dressing of the navel; following the plan usually pursued, that is, folding a piece of linen three or four times, making a hole in it for umbilical cord, then applying bandage three inches wide, and three or four feet long, drawing it pretty tightly, so as to give sufficient support to abdomen, passing it around three or four times. This child never had an attack of *fits*, as they termed it, and is now in fine health. In a few weeks I had another opportunity, which resulted, under the same management, with equal success. Previous to this there had been some fifteen or twenty deaths from this disease on this plantation. I have every reason to believe that it depends upon the manner in which the abdominal bandage is applied.

The dressing of the infant is generally—I might say always —left to some female present, and for fear of *hurting the navel*, she applies the bandage too loosely; the consequences are, when the child cries, the umbilicus is protruded, in some cases, slight umbilical hernia, and otherwise irritating the navel, although it may externally appear perfeclly cicatrized.

The navel should not be troubled or examined under four days, after removing it: the bandage should again be applied, and worn for three or four weeks, or as many months would not be amiss.

It has been supposed by some that the disease has been produced by using old rusty scissors to divide the cord: this I am confident is not so, having known the nicest instruments used with no better success.

I hope physicians having an opportunity of testing this, will give it their attention. Respectfully,

F. M. Fitzhugh.

Madison county, Miss., Jan. 20, 1846.

---

Lowndesboro', Ala., March 20th, 1846.

*Messrs. Editors:* After stating the mortality of New Or leans in 1845, you remark concerning *Trismus Nascentium*, that "this affliction is much more common here, than any where else in the United States, and demands careful investigation."

After assuring you that the result of such "careful investigation" would form a chapter in your Journal of the highest interest to me, at least, I must beg your indulgence in the expression of a doubt of the correctness of your opinion in regard to the disease being more common in New Orleans than any where else in the United States. It is a disease of fearful frequency on the cotton plantations in this section of Alabama. I am not prepared to compare it with other maladies in respect to frequency, but I believe that it destroys more negroes than any other single disease, in this region of country. In a practice of ten years amongst these plantations, I have seen a great many cases. Sometimes, I have found it of such frequent occurrence as to present the appearance of an epidemic. Yet *I have never seen a white child afflicted with the disease.* Is this the case in New Orleans?

Again: I have never seen a case of *decided* Trismus Nascentium that did not prove fatal. Indeed, so well is this characteristic of the disease now known, that it is very generally deemed utterly useless to call in medical aid, after the initiatory symptoms are well developed.

I have tried every plan of treatment which books, or the most anxious study on my part could suggest, but all wholly in vain.

I have made post-mortem examinations in several cases, and found the pathological appearances as uniform as in any other disease. They are as follows:—Heavy vascular engorgement of the peritoneum throughout its whole extent, denoting the highest inflammation. All the portion surrounding the entrance of the umbilical cord into the abdomen, for a circumference of from one to three inches, was in a gangrenous condition. The liver was unnaturally heavy and stiff, with its veins fully injected with fluid blood. There was also heavy engorgement of the substance and membranes of the base of the brain, and along the medulla oblongata, and cervical portion of the spinal marrow.

I have usually observed the first symptoms to make their appearance about the time the umbilical cord comes away, and from this I at first supposed that it was the effect of awkwardness in dressing the navel by the ignorant midwives who usually attend on the plantations, but careful investigation led to nothing conclusive on this point.

Having taken the liberty to offer you these few facts, under the hope that they will not be entirely useless to you, allow me to subscribe myself,

Your obedient servant,

H. V. Wooten, M. D.

